# Supplementary material for: Spontaneous EBV-Reactivation during B Cell Differentiation as a Model for Polymorphic EBV-Driven Lymphoproliferation
Source: Cancers (Basel). 2023 Jun 7;15(12):3083. doi: 10.3390/cancers15123083 (PMC10296496; doi:10.3390/cancers15123083)
Supplement: Supplementary file 1 [file cancers-15-03083-s001.zip › EBV_Cancers_SupplementalData/Supplemental Figures/Supplemental Figure 9_L.pdf]

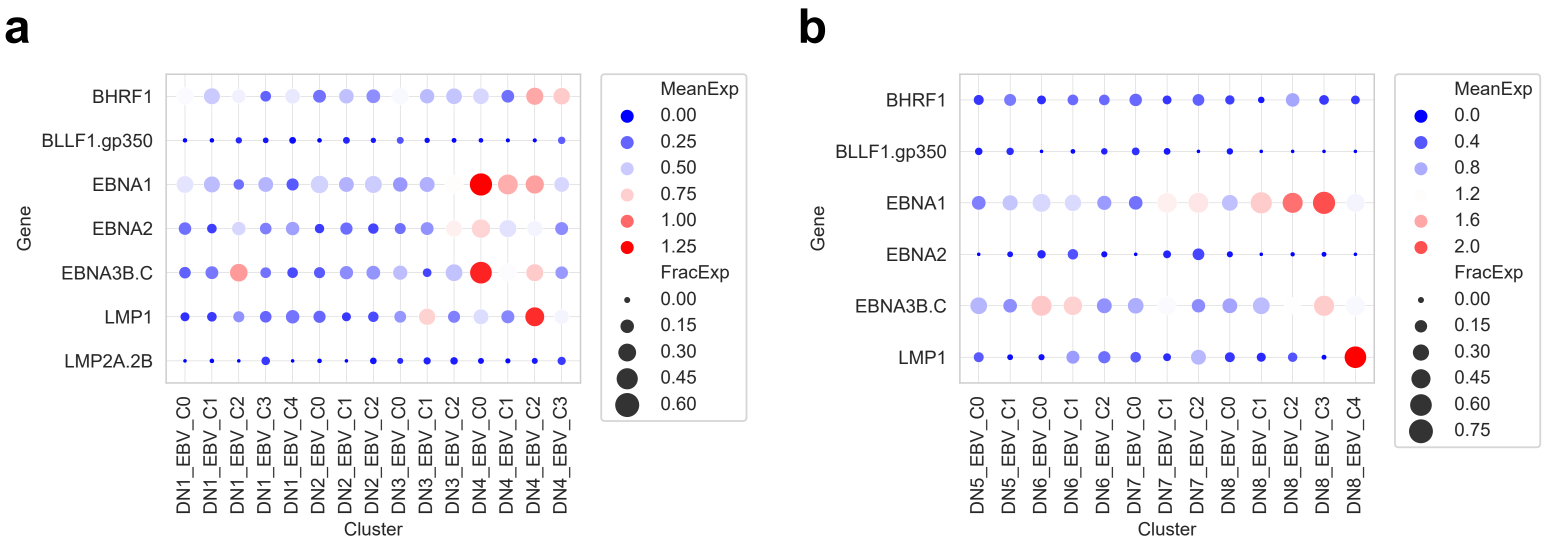

Supplemental Figure 9. EBV gene expression across donor level cell clusters: the bubble-plot illustrates the pattern of EBV gene expression observed across the donor level cell clusters a) in experiment 1 and b) experiment 2. EBV gene identities are identified on the left, and cluster identities along the lower edge of each plot. Mean gene expression is illustrated on a normalised scale across the cell cluster (blue 0.00 to red 1.25) and fraction of cells in the cluster with expression is illustrated by size as indicated in the legend.
