# Supplementary material for: Spontaneous EBV-Reactivation during B Cell Differentiation as a Model for Polymorphic EBV-Driven Lymphoproliferation
Source: Cancers (Basel). 2023 Jun 7;15(12):3083. doi: 10.3390/cancers15123083 (PMC10296496; doi:10.3390/cancers15123083)
Supplement: Supplementary file 1 [file cancers-15-03083-s001.zip › EBV_Cancers_SupplementalData/Supplemental Figures/Supplemental Figure 8_L.pdf]

a

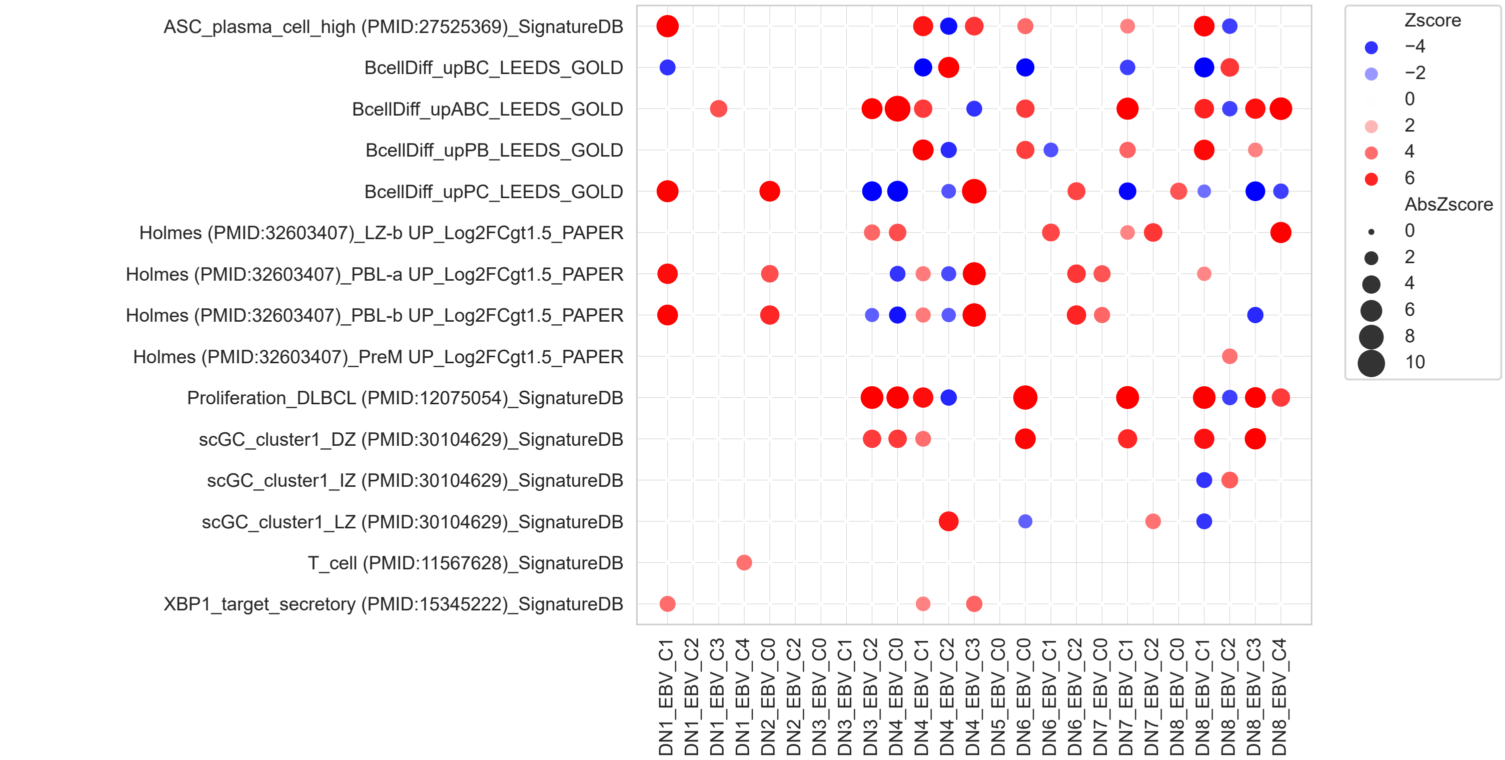

b

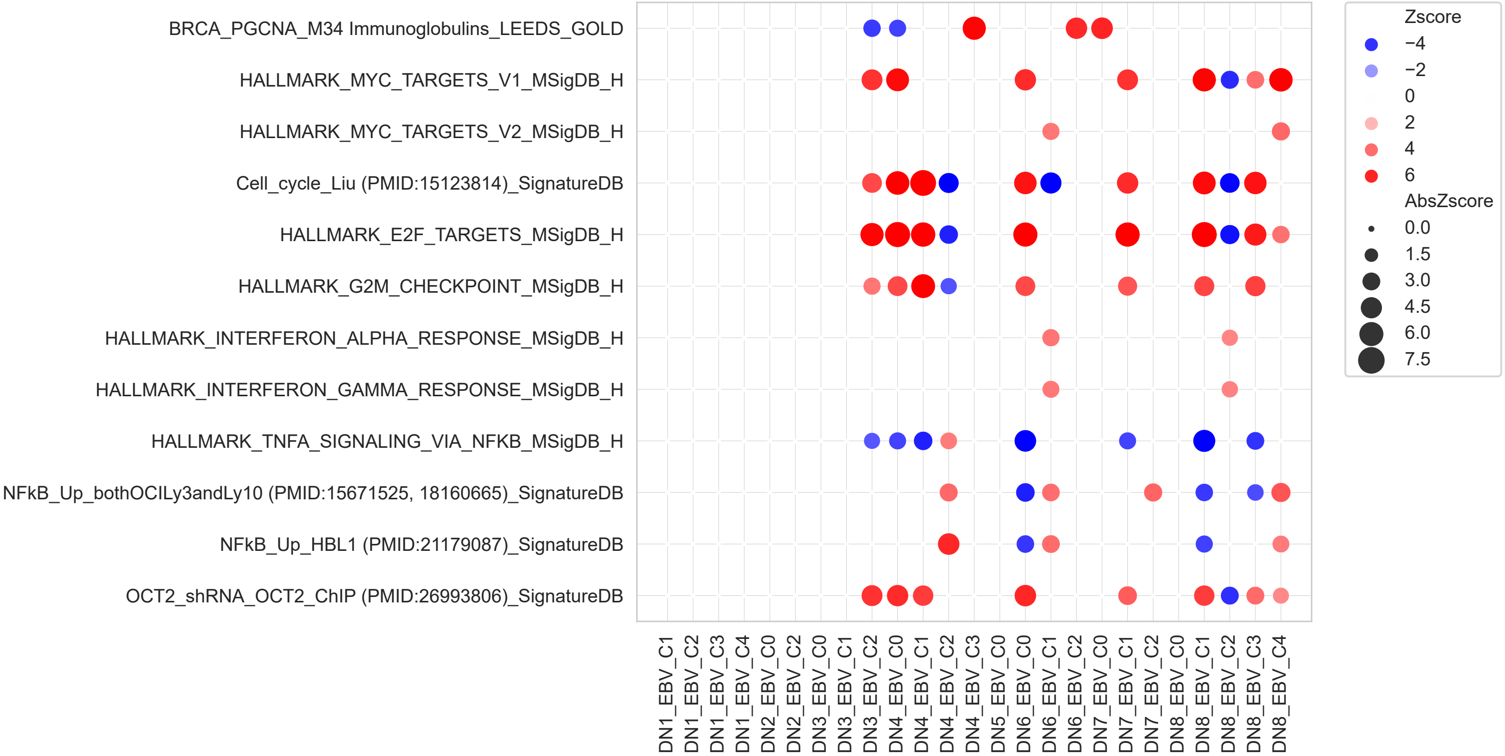

Supplemental Figure 8. Select signature enrichments for EBV-associated cell clusters at individual donor level: the bubble-plot illustrates select signature enrichments across the identified cell clusters all donors (DN1-DN8), upper part of figure illustrates signature terms relate to B-cell differentiation states, lower part of figure illustrates signatures related to selected pathways as indicated on the left of the figure. Significant enrichment is illustrated on a blue to red colour scale (Z-score) with bubble size reflecting FDR significance.
