# Supplementary material for: Spontaneous EBV-Reactivation during B Cell Differentiation as a Model for Polymorphic EBV-Driven Lymphoproliferation
Source: Cancers (Basel). 2023 Jun 7;15(12):3083. doi: 10.3390/cancers15123083 (PMC10296496; doi:10.3390/cancers15123083)
Supplement: Supplementary file 1 [file cancers-15-03083-s001.zip › EBV_Cancers_SupplementalData/Supplemental Figures/Supplemental Figure 2_L.pptx]

## Slide 1
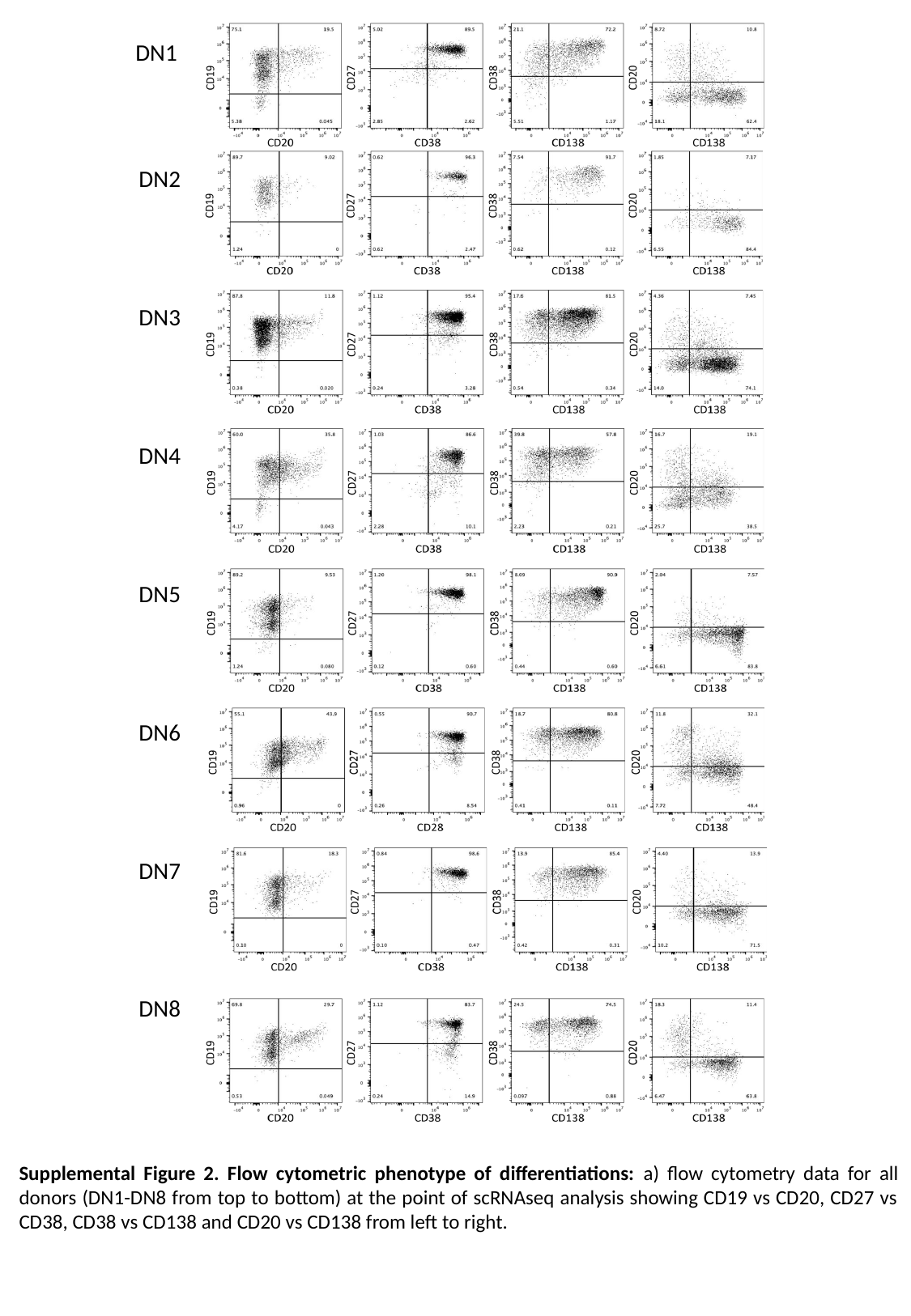

DN1
DN2
DN3
DN4
DN5
DN6
DN7
DN8
Supplemental Figure 2. Flow cytometric phenotype of differentiations: a) flow cytometry data for all donors (DN1-DN8 from top to bottom) at the point of scRNAseq analysis showing CD19 vs CD20, CD27 vs CD38, CD38 vs CD138 and CD20 vs CD138 from left to right.
