# Supplementary material for: Spontaneous EBV-Reactivation during B Cell Differentiation as a Model for Polymorphic EBV-Driven Lymphoproliferation
Source: Cancers (Basel). 2023 Jun 7;15(12):3083. doi: 10.3390/cancers15123083 (PMC10296496; doi:10.3390/cancers15123083)
Supplement: Supplementary file 1 [file cancers-15-03083-s001.zip › EBV_Cancers_SupplementalData/Supplemental Figures/Supplemental Figure 6_L.pdf]

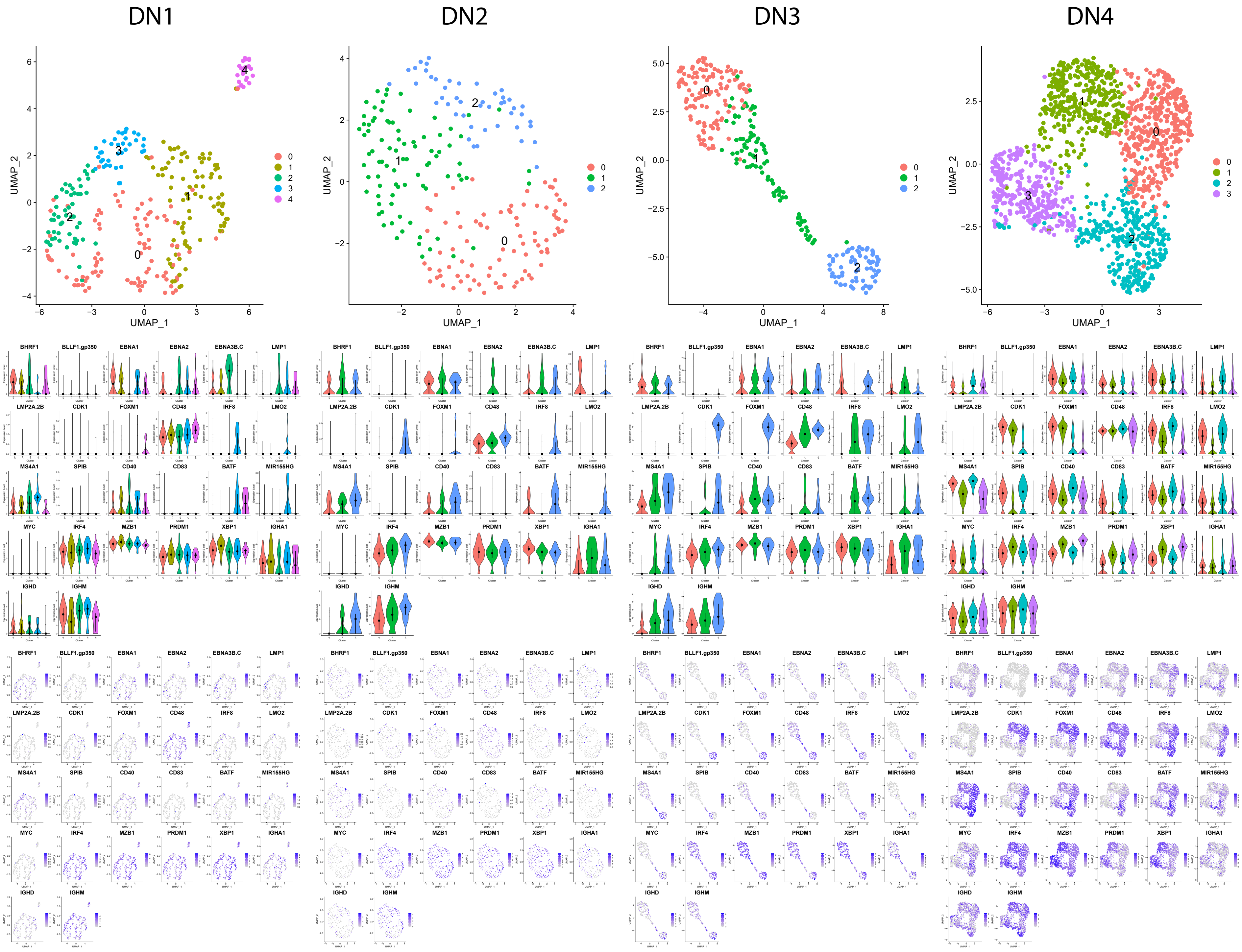

Supplemental Figure 6. Donor level analysis for EBV-associated cells in experiment 1: Analysis of EBV-associated cells for individual donors shown for DN1-4 from left to right, for each divided to show the UMAP plot with cell clusters identified with colour coding, beneath this are shown patterns of index gene expression with each gene shown identified by official gene symbol and plotted either as violin plots of gene expression divided by cell clusters (middle panels) or expression mapped onto individual cells in the UMAP plots (lower panels).
