# Supplementary material for: Spontaneous EBV-Reactivation during B Cell Differentiation as a Model for Polymorphic EBV-Driven Lymphoproliferation
Source: Cancers (Basel). 2023 Jun 7;15(12):3083. doi: 10.3390/cancers15123083 (PMC10296496; doi:10.3390/cancers15123083)
Supplement: Supplementary file 1 [file cancers-15-03083-s001.zip › EBV_Cancers_SupplementalData/Supplemental Figures/Supplemental Figure 5_L.pptx]

## Slide 1
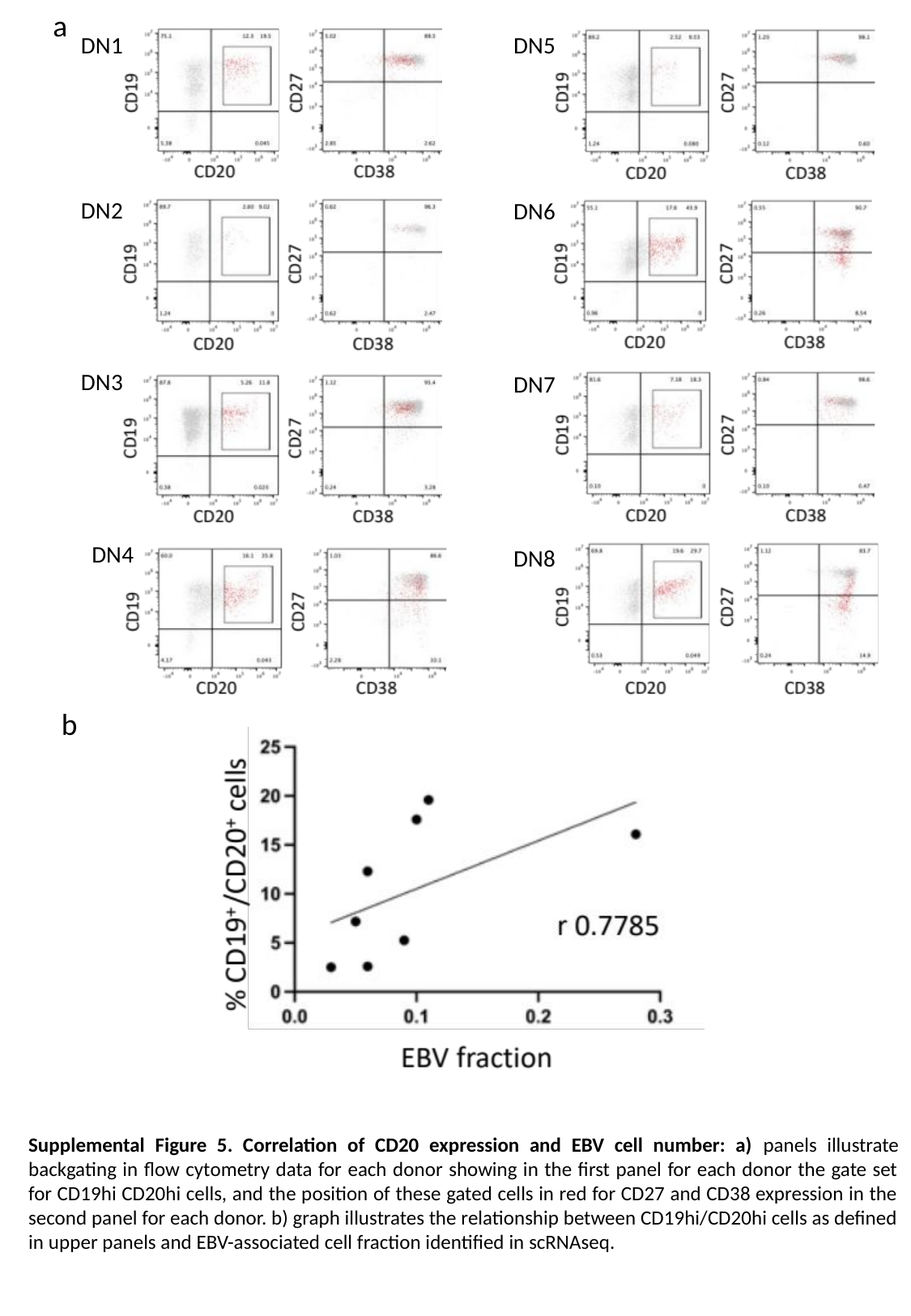

a
DN5
DN1
DN2
DN6
DN3
DN7
DN4
DN8
b
Supplemental Figure 5. Correlation of CD20 expression and EBV cell number: a) panels illustrate backgating in flow cytometry data for each donor showing in the first panel for each donor the gate set for CD19hi CD20hi cells, and the position of these gated cells in red for CD27 and CD38 expression in the second panel for each donor. b) graph illustrates the relationship between CD19hi/CD20hi cells as defined in upper panels and EBV-associated cell fraction identified in scRNAseq.
