# Supplementary material for: Spontaneous EBV-Reactivation during B Cell Differentiation as a Model for Polymorphic EBV-Driven Lymphoproliferation
Source: Cancers (Basel). 2023 Jun 7;15(12):3083. doi: 10.3390/cancers15123083 (PMC10296496; doi:10.3390/cancers15123083)
Supplement: Supplementary file 1 [file cancers-15-03083-s001.zip › EBV_Cancers_SupplementalData/Supplemental Figures/Supplemental Figure 4_L.pdf]

a

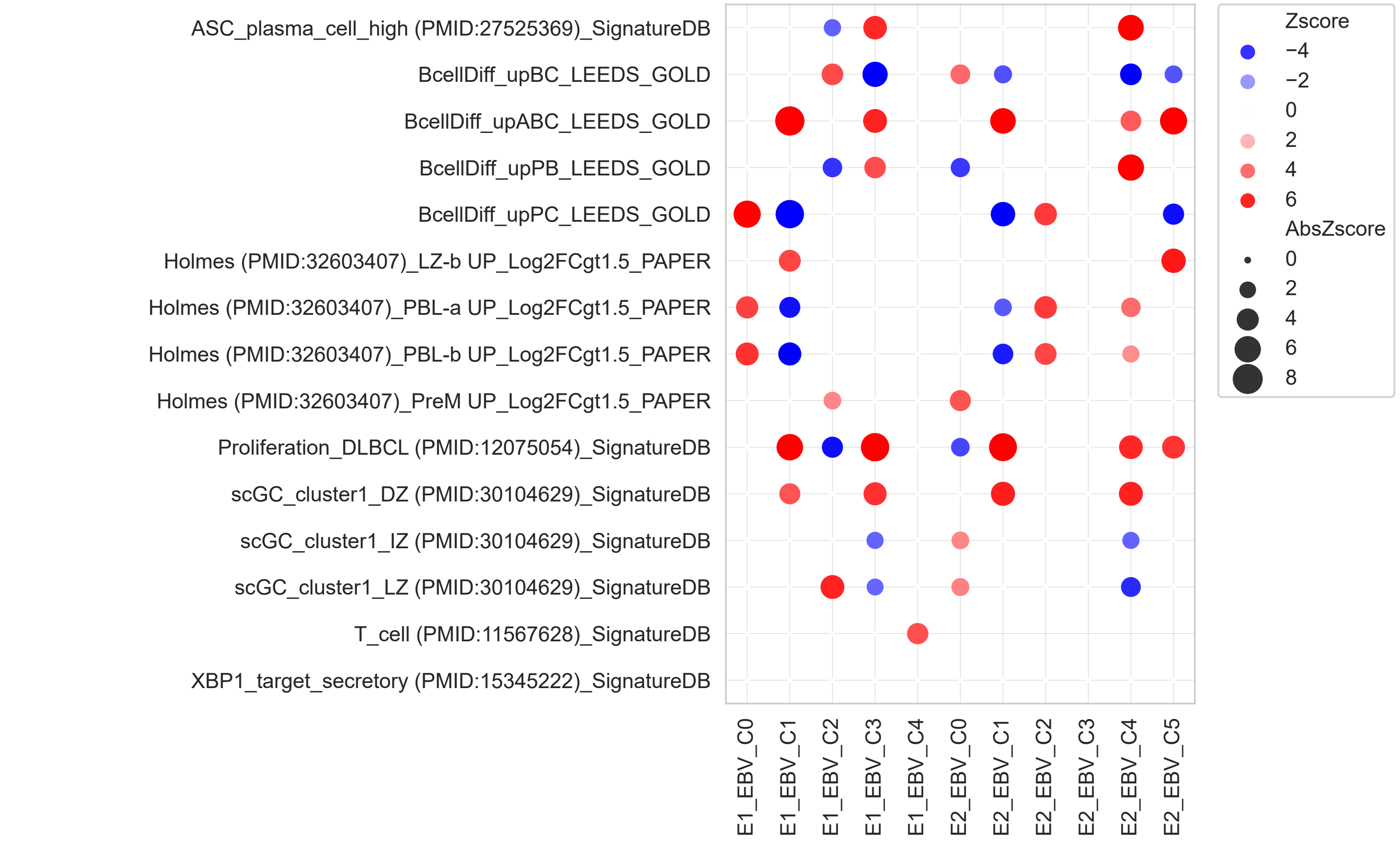

b

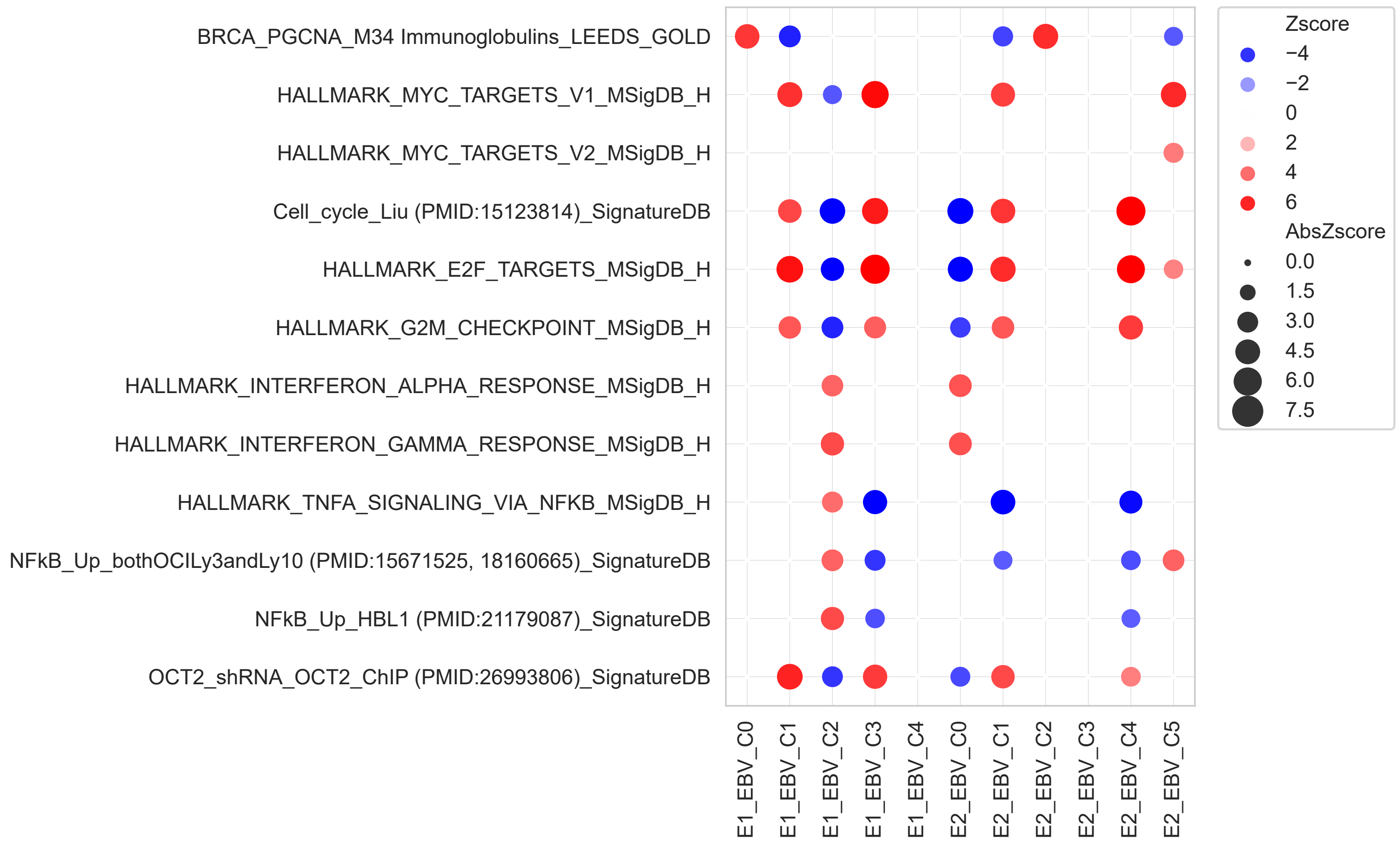

Supplemental Figure 4. Select gene signature associations for EBV-associated cell clusters in E1 and E2: the bubble-plot illustrates select signature enrichments across the identified cell clusters for E1 and E2, upper part of figure illustrates signature terms related to B-cell differentiation states, lower part of figure illustrates signatures related to selected pathways as indicated on the left of the figure. Significant enrichment is illustrated on a blue to red colour scale (Z-score) with bubble size reflecting FDR significance.
