# Supplementary material for: Spontaneous EBV-Reactivation during B Cell Differentiation as a Model for Polymorphic EBV-Driven Lymphoproliferation
Source: Cancers (Basel). 2023 Jun 7;15(12):3083. doi: 10.3390/cancers15123083 (PMC10296496; doi:10.3390/cancers15123083)
Supplement: Supplementary file 1 [file cancers-15-03083-s001.zip › EBV_Cancers_SupplementalData/Supplemental Figures/Supplemental Figure 1_L.pptx]

## Slide 1
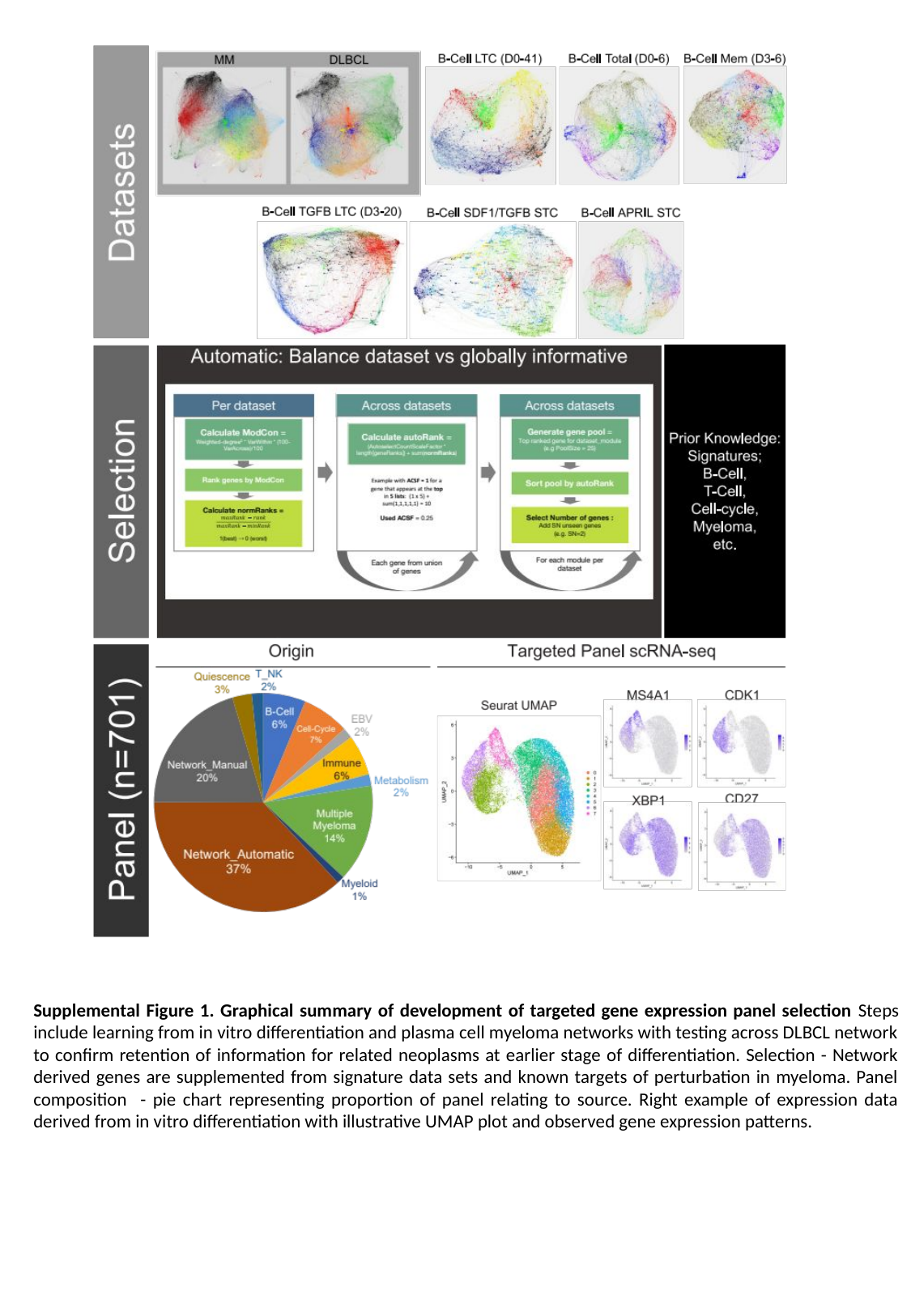

Supplemental Figure 1. Graphical summary of development of targeted gene expression panel selection Steps include learning from in vitro differentiation and plasma cell myeloma networks with testing across DLBCL network to confirm retention of information for related neoplasms at earlier stage of differentiation. Selection - Network derived genes are supplemented from signature data sets and known targets of perturbation in myeloma. Panel composition - pie chart representing proportion of panel relating to source. Right example of expression data derived from in vitro differentiation with illustrative UMAP plot and observed gene expression patterns.
