# Supplementary material for: Spontaneous EBV-Reactivation during B Cell Differentiation as a Model for Polymorphic EBV-Driven Lymphoproliferation
Source: Cancers (Basel). 2023 Jun 7;15(12):3083. doi: 10.3390/cancers15123083 (PMC10296496; doi:10.3390/cancers15123083)
Supplement: Supplementary file 1 [file cancers-15-03083-s001.zip › EBV_Cancers_SupplementalData/Supplemental Figures/Supplemental Figure 3_L.pptx]

## Slide 1
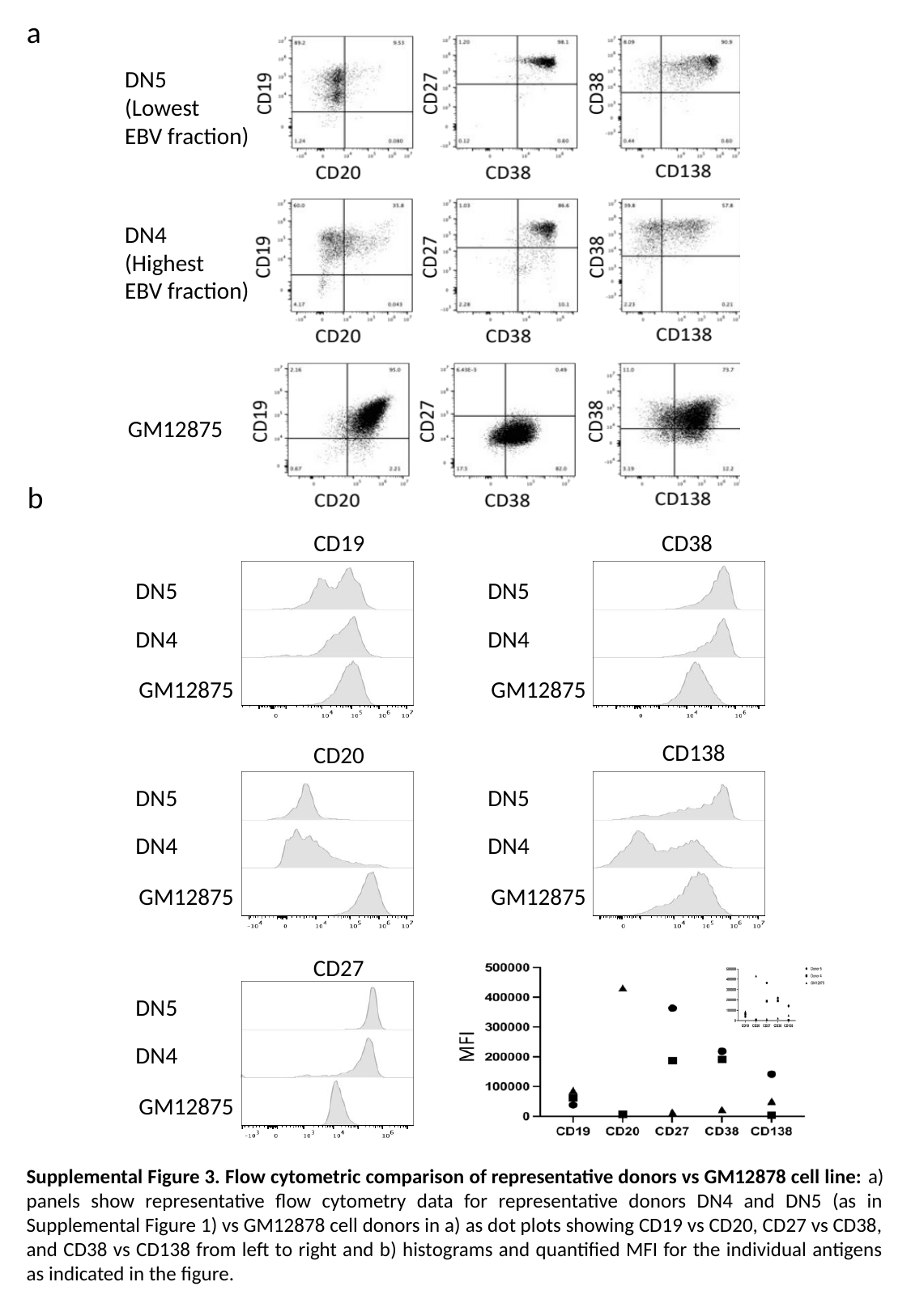

a
DN5
(Lowest
EBV fraction)
DN4
(Highest
EBV fraction)
GM12875
b
CD19
CD38
DN5
DN5
DN4
DN4
GM12875
GM12875
CD138
CD20
DN5
DN5
DN4
DN4
GM12875
GM12875
CD27
DN5
DN4
GM12875
Supplemental Figure 3. Flow cytometric comparison of representative donors vs GM12878 cell line: a) panels show representative flow cytometry data for representative donors DN4 and DN5 (as in Supplemental Figure 1) vs GM12878 cell donors in a) as dot plots showing CD19 vs CD20, CD27 vs CD38, and CD38 vs CD138 from left to right and b) histograms and quantified MFI for the individual antigens as indicated in the figure.
