# Supplementary material for: Spontaneous EBV-Reactivation during B Cell Differentiation as a Model for Polymorphic EBV-Driven Lymphoproliferation
Source: Cancers (Basel). 2023 Jun 7;15(12):3083. doi: 10.3390/cancers15123083 (PMC10296496; doi:10.3390/cancers15123083)
Supplement: Supplementary file 1 [file cancers-15-03083-s001.zip › EBV_Cancers_SupplementalData/Supplemental Figures/Supplemental Figure 10.pptx]

## Slide 1
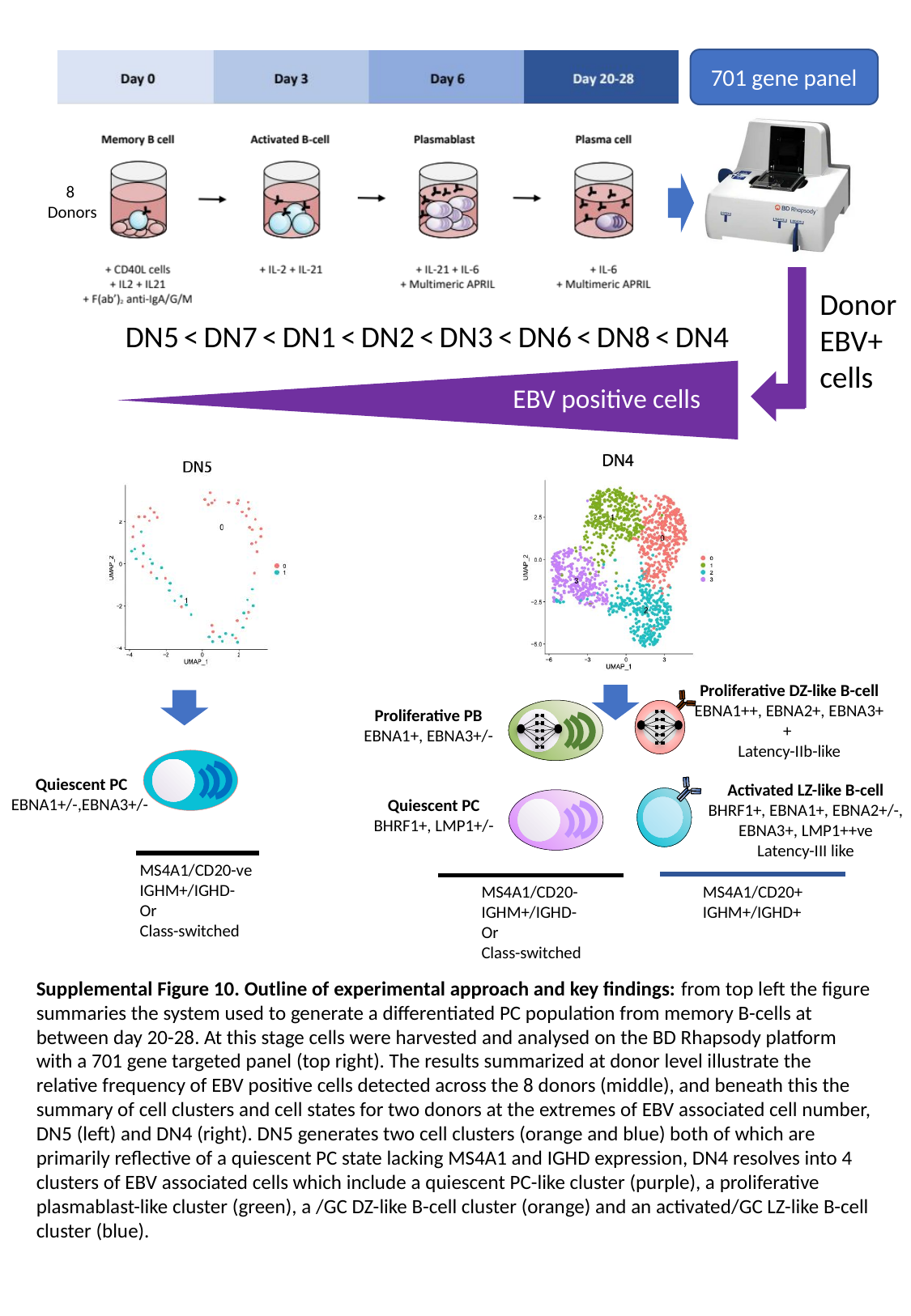

701 gene panel
8
Donors
Donor EBV+ cells
DN5
<
DN7
<
DN1
<
DN2
<
DN3
<
DN6
<
DN8
<
DN4
EBV positive cells
Proliferative DZ-like B-cell
EBNA1++, EBNA2+, EBNA3++
Latency-IIb-like
Proliferative PB
EBNA1+, EBNA3+/-
Quiescent PC
EBNA1+/-,EBNA3+/-
Activated LZ-like B-cell
BHRF1+, EBNA1+, EBNA2+/-, EBNA3+, LMP1++ve
Latency-III like
Quiescent PC
BHRF1+, LMP1+/-
MS4A1/CD20-ve
IGHM+/IGHD-
Or
Class-switched
MS4A1/CD20+
IGHM+/IGHD+
MS4A1/CD20-
IGHM+/IGHD-
Or
Class-switched
Supplemental Figure 10. Outline of experimental approach and key findings: from top left the figure summaries the system used to generate a differentiated PC population from memory B-cells at between day 20-28. At this stage cells were harvested and analysed on the BD Rhapsody platform with a 701 gene targeted panel (top right). The results summarized at donor level illustrate the relative frequency of EBV positive cells detected across the 8 donors (middle), and beneath this the summary of cell clusters and cell states for two donors at the extremes of EBV associated cell number, DN5 (left) and DN4 (right). DN5 generates two cell clusters (orange and blue) both of which are primarily reflective of a quiescent PC state lacking MS4A1 and IGHD expression, DN4 resolves into 4 clusters of EBV associated cells which include a quiescent PC-like cluster (purple), a proliferative plasmablast-like cluster (green), a /GC DZ-like B-cell cluster (orange) and an activated/GC LZ-like B-cell cluster (blue).
